# Decreased gray matter volume and dynamic functional alterations in medicine-free obsessive-compulsive disorder

**DOI:** 10.1186/s12888-023-04740-w

**Published:** 2023-04-25

**Authors:** Zhenning Ding, Zhipeng Ding, Yunhui Chen, Dan Lv, Tong Li, Tinghuizi Shang, Jidong Ma, Chuang Zhan, Xu Yang, Jian Xiao, Zhenghai Sun, Na Wang, Wenbin Guo, Chengchong Li, Zengyan Yu, Ping Li

**Affiliations:** 1 Medical Technology Department, Qiqihar Medical University, Qiqihar, Heilongjiang 161006 China; 2 Department of Psychiatry, Qiqihar Medical University, Qiqihar, Heilongjiang 161006 China; 3 Department of Psychiatry, Baiyupao Psychiatric Hospital of Harbin, Harbin, Heilongjiang 150050 China; 4 Department of Psychiatry, and National Clinical Research Center for Mental Disorders, The Second Xiangya Hospital of Central South University, Changsha, Hunan 410011 China

**Keywords:** Obsessive-compulsive disorder, Gray matter volume, Resting-state, Dynamic functional connectivity

## Abstract

**Background:**

Previous studies discovered the presence of abnormal structures and functions in the brain regions of patients with obsessive-compulsive disorder (OCD). Nevertheless, whether structural changes in brain regions are coupled with alterations in dynamic functional connectivity (dFC) at rest in medicine-free patients with OCD remains vague.

**Methods:**

Three-dimensional T_1_-weighed magnetic resonance imaging (MRI) and resting-state functional MRI were performed on 50 medicine-free OCD and 50 healthy controls (HCs). Firstly, the differences in gray matter volume (GMV) between OCD and HCs were compared. Then, brain regions with aberrant GMV were used as seeds for dFC analysis. The relationship of altered GMV and dFC with clinical parameters in OCD was explored using partial correlation analysis. Finally, support vector machine was applied to examine whether altered multimodal imaging data might be adopted to distinguish OCD from HCs.

**Results:**

Our findings indicated that GMV in the left superior temporal gyrus (STG) and right supplementary motor area (SMA) was reduced in OCD, and the dFC between the left STG and the left cerebellum Crus I and left thalamus, and between the right SMA and right dorsolateral prefrontal cortex (DLPFC) and left precuneus was decreased at rest in OCD. The brain regions both with altered GMV and dFC values could discriminate OCD from HCs with the accuracy of 0.85, sensitivity of 0.90 and specificity of 0.80.

**Conclusion:**

The decreased gray matter structure coupling with dynamic function in the left STG and right SMA at rest may be crucial in the pathophysiology of OCD.

**Trial registration:**

Study on the mechanism of brain network in obsessive-compulsive disorder with multi-model magnetic resonance imaging (registration date: 08/11/2017; registration number: ChiCTR-COC-17,013,301).

**Supplementary Information:**

The online version contains supplementary material available at 10.1186/s12888-023-04740-w.

## Introduction

Obsessive-compulsive disorder (OCD), a mental disorder with intrusive thoughts and/or compulsive behaviour, and affects 2-3% of the general population [1, 2]. However, the pathophysiology of OCD is still unknown. The structural and functional changes associated with OCD have been studied by using high-resolution brain magnetic resonance imaging (MRI) [3, 4].

Voxel-based morphometry (VBM) studies discovered that the gray matter volume (GMV) in the striatum and pallidum was increased, and that in the prefrontal and cingulate cortex was decreased in OCD [5, 6]. In addition, the GMV of the left superior temporal gyrus (STG) was negatively correlated with the severity of OCD [7].

The functional connectivity (FC) can reflect the temporal correlation between distinct brain regions and has been extensively utilized to explore the pathophysiology of mental disorders [8]. However, previous studies assumed that the brain is static and thus ignored the dynamic characteristics of the brain. FC between different brain regions has dynamic properties that changes over time [9], the dynamic FC (dFC) can accurately characterize the cooperation between different brain regions through surveying the time-varying covariance of brain signals at rest [10]. Previous studies have utilized the dFC method to investigate the pathophysiology of OCD. For example, Liu et al. explored dFC changes across brain networks and found that the number of transitions was altered, and positively correlated with clinical symptoms of OCD [11]. Luo et al., who used a similar method, discovered that the fractional time across brain networks was increased, and positively correlated with the anxiety level of OCD [12]. These studies suggested that the dynamic function between different brain regions was altered at rest in patients with OCD [11, 12].

Although most previous studies employed single-modal MRI to investigate the pathophysiology of OCD, the combination of structural and functional analyses may provide new insights into the pathophysiology of mental disorders [13, 14]. Past works discovered the dissociation or overlapping of altered structural and functional MRI features in patients of depressive and bipolar disorder, respectively [15, 16]. Only a few studies have applied multimodal imaging methods to investigate the existence of abnormal structures and functions in the brains of OCD [17–19]. However, previous studies on multimodal neuroimaging were limited to static FC, and whether structural alterations are coupled with changes in dFC in brain regions at rest in OCD remains vague.

In this research, we combined VBM and whole-brain voxel-based dFC methods to explore the brain changes in both structure and dynamic function at rest in medicine-free OCD. Furthermore, we examined the relationship between multimodal MRI alterations and the clinical parameters of OCD. We hypothesized that abnormal GMV coupling with dFC would work together to contribute the pathophysiology of OCD, and could be related with the clinical characteristics of OCD. We also hypothesized that these altered multimodal MRI characteristics would be utilized as potential biomarkers to identify OCD.

## Methods

### Participants

The Research Ethics Committee of Qiqihar Medical University approved this study, and the study protocol is performed in accordance with the Helsinki Declaration of 2013. All participators and/or their legal guardians signed informed consent forms before participating in the study.

Fifty individuals with OCD (29 males and 21 females) were recruited from Qiqihar Medical University’s Fourth Affiliated Hospital and the Qiqihar Mental Health Centre. Two psychiatrists diagnosed the patients with OCD in accordance with the Structured Clinical Interview for DMS-IV (SCID) patient version. Yale-Brown Obsessive Compulsive Scale (Y-BOCS), Hamilton Anxiety Rating Scale (HAMA) and 17-item Hamilton Rating Scale for Depression (HAMD) were applied to evaluate the severity, anxiety and depression symptoms of OCD, respectively. Patients were included if they had Y-BOCS total score ≥ 16 and HAMD score < 18. All the patients have to be free from any psychotropic medication at least 4 weeks before recruitment. In accordance with the nonpatient version of SCID, we also recruited and screened 50 healthy controls (HCs) (32 males and 18 females) from the community. The following exclusion standards were shared by the participants: (1) serious physical diseases or neurological disorders; (2) a history of alcohol or drug abuse; (3) contraindications for magnetic resonance imaging; and (4) pregnant or breast-feeding women. Moreover, HCs were excluded if they had any first-degree relatives with mental disorders. All participants were 18–45 years old, right-handed and Han Chinese.

### Image scanning parameters and preprocessing

Experimental data were collected with a 3.0-Tesla GE 750 Signa-HDX scanner. The participants were required to wear earplugs to decrease the scanner noise effect. They also were required to relax and to shut their eyes but stay awake. T_1_-weighted images were collected by using a rapid acquisition gradient echo sequence, and the scanning parameters were as follows: 2530 ms TR; 3.39 ms TE; 7° FA; 256 × 192 matrix; 256 × 256 mm^2^ FOV; 1.33 mm/0 mm thickness/interslice gap; and 128 sagittal slices. Resting-state fMRI images were scanned by using an echo-planar imaging sequence with the following setup: 2000 ms TR; 30 ms TE; 3.5 mm/0.6 mm thickness/interslice gap; 200 × 200 mm^2^ FOV; 64 × 64 matrix; 90° FA; 33 axial slices; 240 volumes; and 480 s acquisition time.

The original fMRI data were preprocessed by using the Resting-State fMRI Data Analysis Toolkit (RESTplus) (http://restfmri.net/forum/RESTplus) [20]. The process was as follows: the initial 10 volumes were eliminated; slice timing and head motion (excluding data with translation over 2 mm or rotation over 2°) were corrected; the realigned images were spatially normalized to the Montreal Neurological Institute (MNI) space by applying a new segment to the structural images and resampled to 3 × 3 × 3 mm^3^; a 6 mm isotropic Gaussian kernel was employed for smoothing, covariates (i.e., Friston-24 parameter, cerebrospinal fluid [CSF] and white matter [WM]) were removed, and the data were linearly detrended and filtered to 0.01–0.08 Hz.

### VBM analysis

CAT12 (http://www.neuro.uni-jena.de/cat/) toolbox in SPM12 software package on the MATLAB R2014a (MathWorks, Inc.) platform was adopted for VBM analysis. CAT12 is a critical neuroimaging analytic approach for examining structural changes in local GMV [21]. In addition, it can eliminate operational bias when brain areas are selected and whole-brain measurements are collected [22]. Firstly, the toolbox was used for bias-field correction and noise elimination; skull stripping and gray matter (GM), WM and CSF segmentation. Then, all GM images were spatially normalized to the MNI template by the DARTEL algorithm to obtain images of 1.5 mm^3^ voxels; the data were visually examined. Finally, an 8-mm isotropic Gaussian kernel was used to smooth the normalized GM images.

### DFC analysis

DFC analysis was conducted by utilising RESTplus-based Temporal Dynamic Analysis toolkits. Sliding time-window analysis was adopted to characterize FC temporal dynamics. The minimum window length was required to be greater than or equal to 1/*f*_*min*_ to avoid creating spurious fluctuations in dFC (*f*_*min*_ is the minimum frequency of the time course) [23]. In addition, the window length should not be excessively long for fear that the time-variability of FC is disrupted [24]. Based on previous studies, 50 TRs’ window length was chosen in order to achieve equilibrium between capturing patterns of resting-state fluctuations in dFC and producing credible estimations of correlations between regions [24–26]. The entire time course was divided into 181 windows by using hamming windows with window length = 50 TRs (100 s) and step size = 1 TR (2 s). The brain regions that have been proven to have significant differences in GMV between OCD and HCs were selected as the region of interest (ROI). The seed-based dFC analysis was then performed on each window, i.e., Pearson correlation coefficients were computed between the averaged time course of each ROI and all other voxels in the whole-brain to build a FC map for each window. The FC maps were then improved for normality by applying Fisher’s *r*-to-*z* transformation. For each subject, the standard deviation of FC maps across time windows was computed, which is considered as the summary measure of dFC [27]. The larger standard deviations are indicative of greater fluctuations in FC intensity over time.

### Statistical analysis

SPSS (v. 23.0 Chicago, IL, USA) was utilized for the statistical analysis of demographic and clinical data. If the continuous variables were normally distributed, the two-sample *t* test was performed; otherwise, the Mann-Whitney *U* test was adopted. Categorical data were analyzed by using the chi-square test.

The standard deviation values of FC maps across time windows of all subjects in each group were summarized together to obtain the dFC values at the group level. GMV and dFC values were compared between two groups (OCD *vs* HCs) through voxel-wise two-sample *t*-tests by taking age, gender, education level, total intracranial volume and mean framewise displacement (FD) as covariates. The significant threshold was *P* < 0.05 (Gaussian random field corrected, voxel *P* < 0.001, cluster *P* < 0.05). Finally, we retrieved the mean GMV and dFC values from brain areas with significant inter-group differences and conducted a partial correlation analysis with clinical parameters (i.e., disease duration, Y-BOCS total and subscale scores, HAMA and HAMD scores). Age, gender and education level were controlled as covariates, and the significance level was set at *P* < 0.05 (Bonferroni adjusted).

### Support vector machine for classification analysis

Support vector machine (SVM) is widely applied in classification because of its ability to process high-dimensional data and high classification accuracy [28]. In this study, we performed SVM analysis on the basis of the LIBSVM package in MATLAB to determine whether changed GMV and dFC can discriminate between OCD and HCs. Brain regions with altered GMV and dFC values were input into the classification model as feature variables. All the sample data were divided into a training set and a test set. The training set was employed to train the SVM classifier, and the test set was adopted to evaluate classification performance. Linear kernel was used to reduce the risk of data overfitting. Considering the small sample size, we adopted “leave-one-out” cross-validation (LOOCV) to verify the classifier’s capacity to discriminate between two groups, and acquired the greatest sensitivity and specificity values. This operation was repeated for each sample for the purpose of obtaining the total accuracy of SVM. The permutation test was at a repetition of 5,000 times in order to evaluate the statistical significance of classification accuracy. Finally, the receiver operating characteristic (ROC) curve was generated to demonstrate the performance of the SVM model.

### Validation analyses

Based on the parameter settings in previous studies, we used different window lengths (i.e., 30 TRs [60 s], 80 TRs [160 s]) and step sizes (i.e., 3 TRs [6 s] and 5 TRs [10 s]) to exclude the influence of parameter selection and to verify dFC stability [29–32]. Meanwhile, the static FC map of each ROI was calculated and compared voxel-wise between OCD and HCs.

## Results

### Demographics and clinical data

No significant differences were found in age, gender, education and mean FD between the two groups. Y-BOCS total, obsessive and compulsive subscale scores, HAMD and HAMA scores showed significant inter-group differences (Table 1).

**Table 1:** Demographic and clinical characteristics of participants

Variables	OCD (n = 50) Mean ± SD	HCs (n = 50) Mean ± SD	*t*/χ^2^/*U*	*P*-value
Age (years)	26.36 ± 7.97	25.60 ± 7.94	0.478^a^	0.634
Gender (male/female)	29/21	32/18	0.378^b^	0.539
Education (years)	13.16 ± 2.92	12.38 ± 3.03	1.312^a^	0.193
Illness duration (months)	64.04 ± 72.58	--	--	--
Y-BOCS				
Total	25.36 ± 6.07	1.02 ± 0.87	28.052^a^	< 0.001^*^
Obsessive subscale score	13.04 ± 4.28	0.34 ± 0.48	20.871^a^	< 0.001^*^
Compulsive subscale score	12.32 ± 4.30	0.66 ± 0.69	18.924^a^	< 0.001^*^
HAMD	8.60 ± 4.34	1.38 ± 0.97	11.488^a^	< 0.001^*^
HAMA	11.06 ± 7.03	1.02 ± 0.96	10.004^a^	< 0.001^*^
FD	0.07 ± 0.02	0.07 ± 0.04	1133.000^c^	0.420

Data was displayed with mean ± standard deviation

a. Two sample *t*-test

b. Pearson chi-square

c. Mann-Whitney *U* test

*. Significant difference

Abbreviations: OCD = obsessive-compulsive disorder; HCs = healthy controls; Y-BOCS = Yale-Brown Obsessive-Compulsive Scale; HAMD = 17-item Hamilton Depression Rating Scale; HAMA = Hamilton Anxiety Rating Scale; FD = framewise displacement

### VBM results

**Fig. 1:**
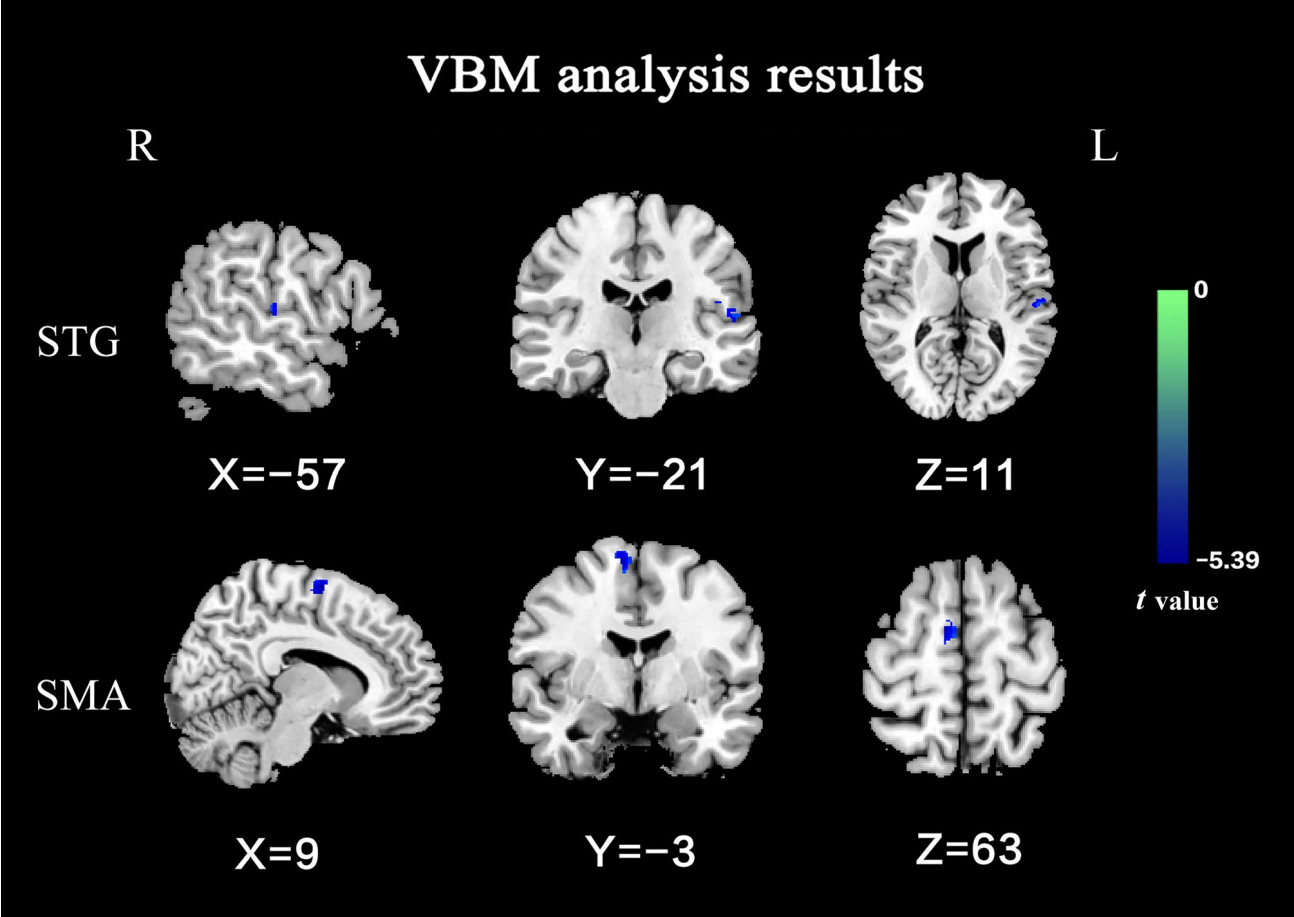
Brain regions with significant differences on GMV between OCD and HCs. The color bar represents the *t* values from the two-sample *t*-tests. Blue color denotes decreased GMV in OCD. STG: superior temporal gyrus; SMA: supplementary motor area; GMV: gray matter volume; OCD: obsessive-compulsive disorder; HCs: healthy controls

Compared with the HCs, OCD exhibited significantly decreased GMV in the left STG and right supplementary motor area (SMA) (Table 2; Fig. 1).

**Table 2:** Brain regions with abnormal gray matter volume in OCD

Cluster location	Peak (MNI)			Cluster size (voxels)	*t* value
	x	y	z		
Left Superior Temporal Gyrus	-57	-21	11	102	-4.381
Right Supplementary Motor Area	9	-3	63	143	-5.394

The significant threshold was *P* < 0.05 (Gaussian random field corrected, voxel *P* < 0.001, cluster *P* < 0.05). Age, gender, and the total intracranial volume were used as covariates to minimize the potential effects of these variables. MNI = Montreal Neurological Institute; OCD = obsessive-compulsive disorder

### DFC results

**Fig. 2:**
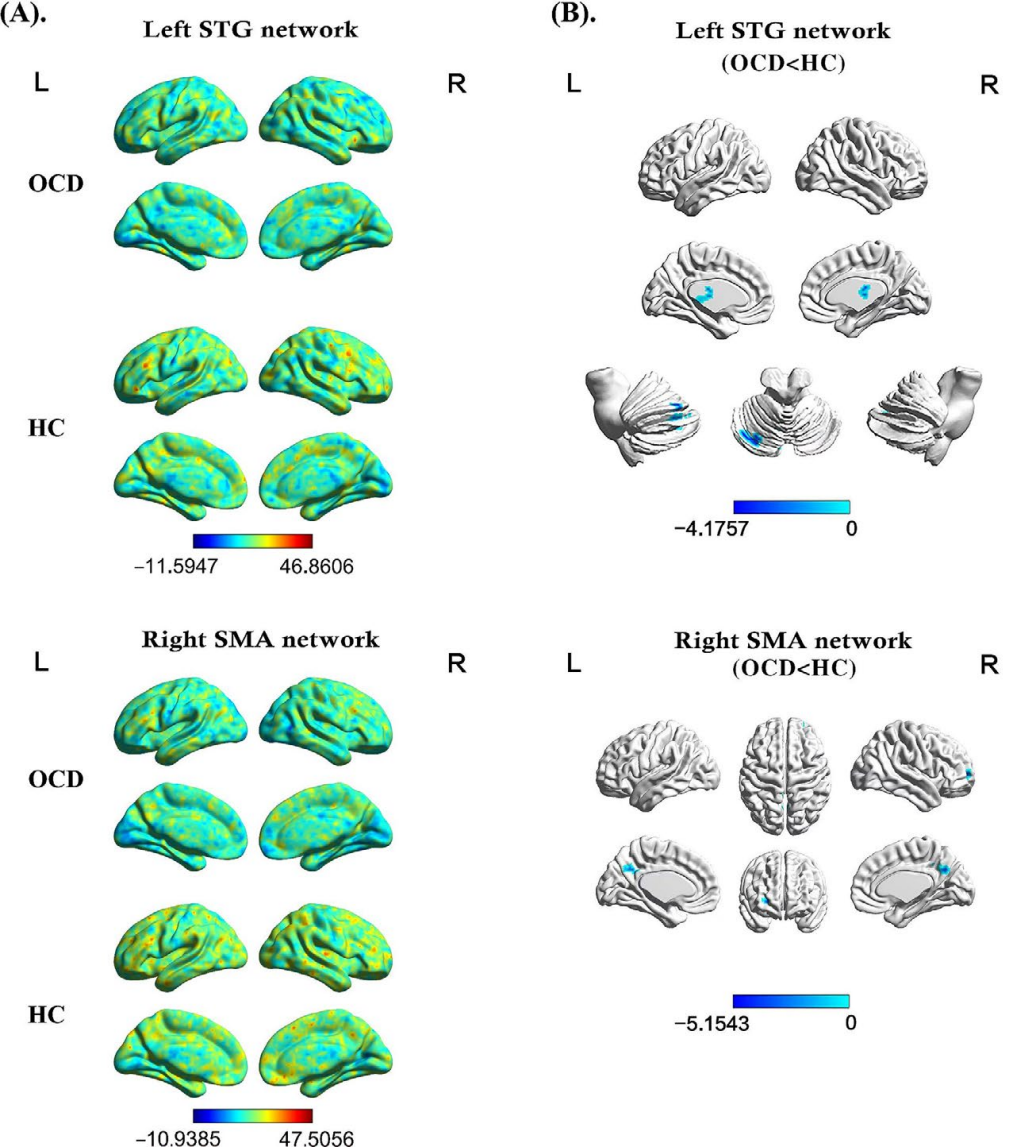
Voxel-wise analysis of dFC patterns in abnormal GMV brain regions. The color bar indicates the *t* values from one/two-sample *t*-tests. (A) DFC pattern maps of the left STG network and right SMA network in OCD and HC group separately. (B) Brain regions with abnormal dFC in OCD. The blue color denotes decreased dFC values in OCD. STG: superior temporal gyrus; SMA: supplementary motor area; dFC: dynamic functional connectivity; OCD: obsessive-compulsive disorder; HC: healthy control; L: left; R: right

OCD displayed significantly decreased dFC between the left STG and the left cerebellum Crus I and left thalamus; and decreased dFC between the right SMA and the dorsolateral prefrontal cortex (DLPFC) and left precuneus (Table 3; Fig. 2).

**Table 3:** Brain regions with abnormal dynamic functional connectivity at rest in OCD

Cluster location	Peak (MNI)			Cluster size (voxels)	*t* value
	x	y	z		
*Seed: left superior temporal gyrus*					
Left Cerebellum Crus I	-3	-75	-33	59	-4.0732
Left Thalamus	3	-18	18	62	-4.1757
*Seed: right supplementary motor area*					
Right DLPFC	24	57	3	23	-5.1543
Left Precuneus	0	-60	36	59	-4.0911

The significant threshold was *P* < 0.05 (Gaussian random field corrected, voxel *P* < 0.001, cluster *P* < 0.05). Age, sex, and the mean FD values were used as covariates to minimize the potential effects of these variables. MNI = Montreal Neurological Institute; DLPFC = dorsolateral prefrontal cortex; OCD = obsessive-compulsive disorder; FD = framewise displacement

### Correlations between GMV and dFC values and clinical variables

No correlations were found between decreased GMV, dFC and clinical variables (i.e., Y-BOCS total and subscale scores, HAMA and HAMD scores and illness duration) in OCD.

### SVM results

**Fig. 3:**
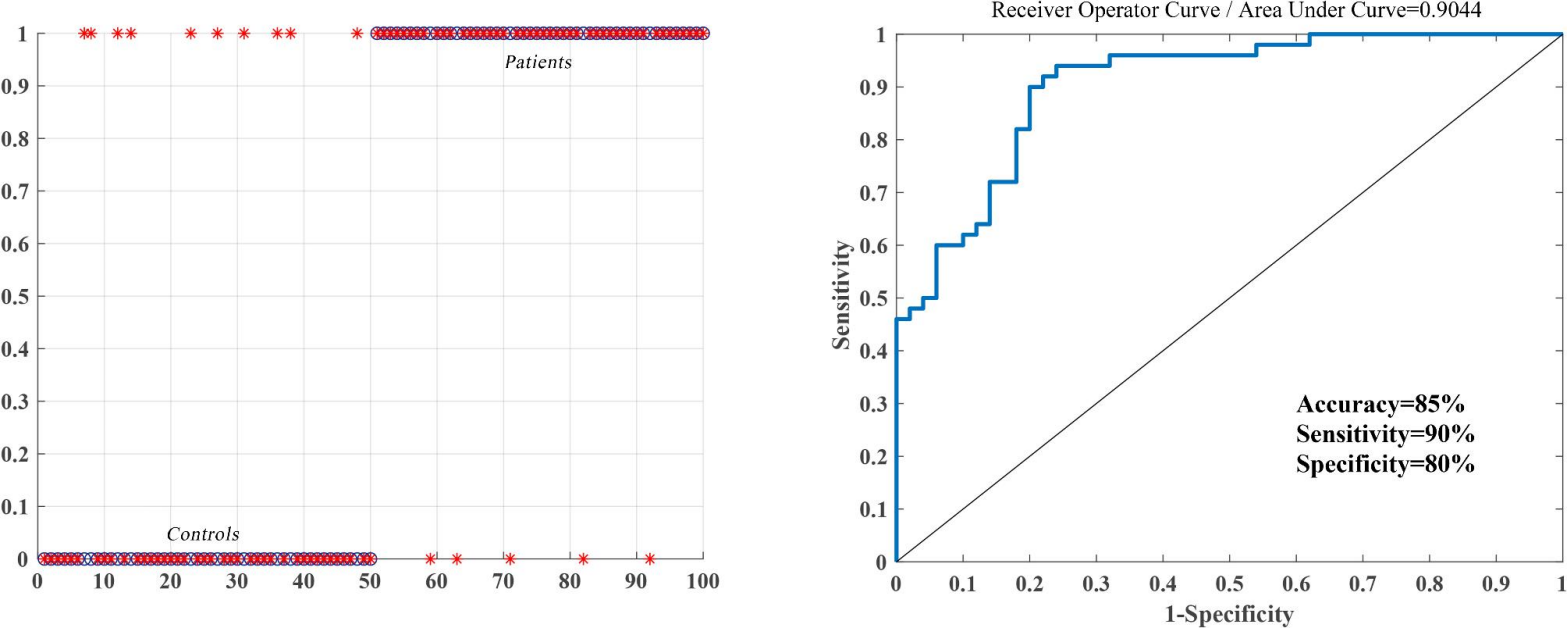
Classification performance for OCD and HCs combined with GMV and dFC results (features A, B, C, D, E, and F). Left: Classification plots for the SVM classifier. Right: ROC curves assessing SVM performance. SVM: support vector machine; GMV: gray matter volume; dFC: dynamic functional connectivity; OCD: obsessive-compulsive disorder; HCs: healthy controls

Six feature variables (A = GMV of left STG; B = GMV of right SMA; C = dFC of left STG-left cerebellum Crus I; D = dFC of left STG-left thalamus; E = dFC of right SMA-right DLPFC; F = dFC of right SMA-left precuneus) were entered into the classification models. The area under the curve (AUC), accuracy, specificity and sensitivity for each feature are summarized in Supplementary Table S1 and Fig. S1. The combination of six brain regions both with altered GMV and dFC values (features A, B, C, D, E and F) could differentiate OCD from HCs with an accuracy of 0.85, sensitivity of 0.90 and specificity of 0.80 (*P* < 0.001, nonparametric permutation test). In addition, the AUC of the ROC curve that was used to verify the performance of SVM was 0.9044 (Fig. 3).

### Validation analyses

The results of dFC between two groups with diverse sliding window lengths and step sizes were similar to those of dFC with sliding window length = 50 TRs and step size = 1 TR (Supplementary Tables S2–S5 and Fig. S2–S5). Moreover, we found no differences in static FC between OCD and HCs (Fig. S6).

## Discussion

In the present research, we employed VBM and dFC methods to investigate both the structural and dynamic functional alterations in brain regions at rest in OCD. The GMV values of the left STG and right SMA were reduced; the dFC values between the left STG and left cerebellum Crus I and left thalamus, and between the right SMA and right DLPFC and left precuneus were decreased at rest in OCD relative to those in HCs. The combination of brain regions with altered GMV and dFC values could be utilized to identify OCD. Our present results demonstrated that the brain regions with structural alterations are accompanied by the dynamic function changes at rest in OCD.

Static FC reflects the average strength of connectivity in different brain regions over time, and cannot describe the whole process of spontaneous neural activities in the brain at rest [33]. DFC is evaluated as the time-varying covariance of neural signals between brain regions at rest, which can describe the cooperation between brain regions in a precise way, and reflect the degrees and patterns of connectivity [10, 33]. The decrease of dFC may indicate the obstacle of dynamic functional integration within or between brain networks, which is manifested as the abnormal spontaneous and/or recurrence patterns of connectivity [34].

The current research discovered reduced GMV in the left STG, and decreased dFC values between the left STG and left cerebellum Crus I and left thalamus at rest in medicine-free OCD. Consistent with our work, a previous study discovered reduced GMV in the left STG in patients with OCD [35]. STG has been found to be involved in modulation of the reward processing and emotional information [36]. The reduced GMV of the left STG may participate in the pathophysiology of OCD via the roles of STG in reward and emotional processing. Moreover, reduced GMV may be a manifestation of the long-lasting and highly stable change of the disease [37]. For this reason, we infer that the defect in the processing of rewards and emotion may be the stable and lasting clinical symptom of OCD [38].

The cerebellum is involved in cognitive control and information processing [39, 40]. The thalamus plays a key role in perception and thoughts integration, motor and executive function [41, 42]. Previous studies reported reduced regional homogeneity (ReHo) in the cerebellum and thalamus at rest in OCD [43, 44]. Moreover, the decreased dynamic amplitude of the low-frequency fluctuation in the cerebellum was discovered in OCD [45]. The decreased dFC values between the left STG and left cerebellum Crus I-left thalamus at rest may reflect a malfunction of functional integration between these brain regions in OCD at the time [37].

In addition, we found reduced GMV in the right SMA, and decreased dFC values between the right SMA and right DLPFC and left precuneus at rest in OCD. SMA is a part of the sensorimotor cortico-striato-thalamo-cortical (CSTC) circuit and is involved in action selection and habitual behaviour [46, 47]. A previous meta-analysis discovered low ReHo in the SMA at rest in OCD [44]. Our current and previous results suggested that the reduced GMV and low local spontaneous neural activity of the SMA may work together to contribute the pathophysiology of OCD.

As an important part of dorsal cognitive CSTC circuits, DLPFC is associated with executive functions (e.g., response inhibition and planning) [47]. Previous works illustrated the key role of DLPFC in the pathophysiology of OCD [48–50]. In our previous study, we found decreased degree values of the DLPFC at rest in OCD [51]. The precuneus is a crucial component of the default-mode network (DMN) and participates in self-awareness processing [52–54]. Additionally, abnormal GMV and function in the precuneus at rest have also been found in OCD [35, 55–57]. The decreased dFC values between the right SMA and right DLPFC and left precuneus at rest discovered in the present work may imply the dysfunction of dynamic functional integration between the sensorimotor and dorsal cognitive CSTC circuits and DMN, and may be related to the insufficient inhibition ability for self-awareness and habitual behaviour at rest in OCD [46–50, 57].

The SVM analysis revealed that the combination of altered GMV and dFC in the left STG, right SMA, left cerebellum Crus I, left thalamus, right DLPFC and left precuneus were able to distinguish the OCD from HCs with an accuracy of 0.85, sensitivity of 0.90 and specificity of 0.80. This result suggested that altered multimodal MRI characteristics perform an essential role in the pathogenesis and classification of OCD.

Validation analysis revealed that the current findings were not dependent on parameter selection (i.e., window length and step size) and had good reproducibility. Moreover, static FC did not show differences between the two groups, indicating that dFC may be utilized to describe voxel-wise FC alterations within a shorter time scale and is more sensitive than static FC [29].

Inconsistent with our hypothesis, reduced GMV and dFC were not correlated with clinical parameters in OCD. Prior studies also discovered that changed structure and function had no correlations with clinical parameters in OCD [58–61]. Therefore, we inferred that the changed GMV and dFC may be a trait alteration of OCD and not dependent on the current clinical status [59, 60]. However, the GMV of the left STG was negatively correlated with the severity of OCD [7]. The heterogeneity of OCD, relatively small sample sizes, and rigorous Bonferroni correction might limit the relationship between abnormal multimodal MRI characteristics and clinical factors in OCD, and may explain this contradiction [62, 63].

The current study has some limitations. Firstly, there has no consensus on the optimal window length of sliding-window method, and the selection of different window lengths may have an impact on the results, but our validation analysis using different window sizes suggests that our findings are stable and not substantially influenced by the choice of sliding window length. Secondly, dFC analysis is especially sensitive to head motion [64]. Although mean FD values were regressed in statistical analyses and had no group differences, head motion remains a possible source of artifacts. Thirdly, the current results were not tested on another independent sample, which may lead to the overfitting of the SVM results. Finally, whether the brain regions both with abnormal GMV and dFC discovered in this study will change with the intervention on OCD needs to be investigated in longitudinal follow-up studies.

## Conclusion

We combined the VBM and dFC methods to investigate structural and dynamic functional alterations simultaneously in medicine-free patients with OCD, and discovered that the GMV abnormalities in the left STG and right SMA were accompanied by dFC changes at rest in OCD. Moreover, a combination of brain regions both with reduced GMV and dFC could be used to identify OCD. The current findings highlight the crucial role of altered multimodal MRI characteristics in the pathogenesis and classification of OCD.

## Electronic supplementary material

Below is the link to the electronic supplementary material.

Supplementary Material 1 Tables and Figures

## Data Availability

The data may be available from the corresponding author upon reasonable request.

## Abbreviations

OCD: Obsessive–Compulsive Disorder
HCs: Healthy Controls
MRI: Magnetic resonance imaging
dFC: Dynamic functional connectivity
VBM: Voxel-based morphometry
GMV: Gray matter volume
STG: Superior temporal gyrus
SMA: Supplementary motor area
DLPFC: Dorsolateral prefrontal cortex
Y-BOCS: Yale-Brown Obsessive–Compulsive Scale
HAMD: Hamilton Depression Rating Scale
HAMA: Hamilton Anxiety Rating Scale
RESTplus: Resting-State fMRI Data Analysis Toolkit
FD: Framewise displacement
SVM: Support vector machine
ROC: Receiver operating characteristic
AUC: Area under the curve
CSTC: Cortico–striato–thalamo–cortical
