# Supplementary material for: Decreased gray matter volume and dynamic functional alterations in medicine-free obsessive-compulsive disorder
Source: BMC Psychiatry. 2023 Apr 25;23:289. doi: 10.1186/s12888-023-04740-w (PMC10131325; doi:10.1186/s12888-023-04740-w)
Supplement: Supplementary file 1 — Supplementary Material 1 Tables and Figures [file 12888_2023_4740_MOESM1_ESM.docx]

**Supplementary materials**

**Tables S1.** The results of SVM analysis based on the selected features.

| Features | AUC | Accuracy | Sensitivity | Specificity |
| --- | --- | --- | --- | --- |
| A | 0.5572 | 0.57 | 0.68 | 0.54 |
| B | 0.6372 | 0.60 | 0.62 | 0.64 |
| C | 0.8100 | 0.73 | 0.72 | 0.74 |
| D | 0.8064 | 0.73 | 0.70 | 0.76 |
| E | 0.7940 | 0.64 | 0.64 | 1.00 |
| F | 0.6912 | 0.67 | 0.72 | 0.62 |
| AB | 0.6484 | 0.58 | 0.56 | 0.68 |
| AC | 0.8092 | 0.73 | 0.76 | 0.74 |
| AD | 0.8100 | 0.73 | 0.72 | 0.76 |
| AE | 0.8456 | 0.73 | 0.78 | 0.78 |
| AF | 0.7832 | 0.73 | 0.74 | 0.74 |
| BC | 0.8180 | 0.73 | 0.74 | 0.76 |
| BD | 0.8216 | 0.72 | 0.82 | 0.70 |
| BE | 0.8236 | 0.74 | 0.74 | 0.76 |
| BF | 0.7616 | 0.67 | 0.70 | 0.68 |
| CD | 0.8504 | 0.75 | 0.84 | 0.72 |
| CE | 0.8548 | 0.81 | 0.86 | 0.78 |
| CF | 0.8500 | 0.74 | 0.76 | 0.80 |
| DE | 0.8816 | 0.77 | 0.78 | 0.84 |
| DF | 0.8556 | 0.79 | 0.80 | 0.80 |
| EF | 0.8448 | 0.77 | 0.82 | 0.72 |
| ABC | 0.8172 | 0.76 | 0.80 | 0.74 |
| ABD | 0.8168 | 0.75 | 0.84 | 0.70 |
| ABE | 0.8648 | 0.78 | 0.76 | 0.92 |
| ABF | 0.7684 | 0.71 | 0.80 | 0.64 |
| ACD | 0.8404 | 0.71 | 0.70 | 0.82 |
| ACE | 0.8716 | 0.79 | 0.82 | 0.80 |
| ACF | 0.8644 | 0.77 | 0.78 | 0.80 |
| ADE | 0.8824 | 0.80 | 0.74 | 0.90 |
| ADF | 0.8592 | 0.77 | 0.78 | 0.80 |
| AEF | 0.8556 | 0.77 | 0.78 | 0.82 |
| BCD | 0.8664 | 0.80 | 0.88 | 0.74 |
| BCE | 0.8748 | 0.82 | 0.88 | 0.76 |
| BCF | 0.8584 | 0.76 | 0.76 | 0.82 |
| BDE | 0.8976 | 0.81 | 0.82 | 0.84 |
| BDF | 0.8672 | 0.80 | 0.84 | 0.78 |
| BEF | 0.8356 | 0.73 | 0.70 | 0.84 |
| CDE | 0.8888 | 0.79 | 0.84 | 0.84 |
| CDF | 0.8752 | 0.76 | 0.78 | 0.84 |
| CEF | 0.8548 | 0.81 | 0.86 | 0.78 |
| DEF | 0.8904 | 0.83 | 0.88 | 0.78 |
| ABCD | 0.8604 | 0.79 | 0.88 | 0.70 |
| ABCE | 0.8856 | 0.79 | 0.82 | 0.84 |
| ABCF | 0.8544 | 0.76 | 0.76 | 0.84 |
| ABDE | 0.8988 | 0.78 | 0.82 | 0.88 |
| ABDF | 0.8632 | 0.77 | 0.76 | 0.82 |
| ABEF | 0.8476 | 0.74 | 0.72 | 0.86 |
| ACDE | 0.9056 | 0.83 | 0.84 | 0.84 |
| ACDF | 0.8760 | 0.80 | 0.80 | 0.80 |
| ACEF | 0.8820 | 0.81 | 0.86 | 0.78 |
| ADEF | 0.9056 | 0.81 | 0.80 | 0.86 |
| BCDE | 0.9088 | 0.83 | 0.86 | 0.86 |
| BCDF | 0.8800 | 0.79 | 0.80 | 0.80 |
| BCEF | 0.8900 | 0.82 | 0.86 | 0.78 |
| BDEF | 0.8980 | 0.82 | 0.84 | 0.82 |
| CDEF | 0.8964 | 0.80 | 0.84 | 0.82 |
| ABCDE | 0.8720 | 0.77 | 0.80 | 0.86 |
| ABCDF | 0.8864 | 0.81 | 0.90 | 0.74 |
| ABCEF | 0.8808 | 0.79 | 0.88 | 0.74 |
| ABDEF | 0.8684 | 0.80 | 0.82 | 0.82 |
| ACDEF | 0.9000 | 0.82 | 0.84 | 0.82 |
| BCDEF | 0.9016 | 0.84 | 0.90 | 0.80 |
| **ABCDEF** | **0.9044** | **0.85** | **0.90** | **0.80** |

A = GMV of STG; B = GMV of right SMA; C = dFC of left STG-left cerebellum Crus I; D = dFC of left STG-left thalamus; E = dFC of right SMA-right DLPFC; F = dFC of right SMA-left precuneus. STG: superior temporal gyrus; SMA: supplementary motor area; DLPFC: dorsolateral prefrontal cortex; GMV: gray matter volume; dFC: dynamic functional connectivity; AUC: area under curve; SVM: support vector machine.

**Table S2.** Brain regions with abnormal dynamic functional connectivity at rest in OCD (window length = 30 TRs, step size = 1 TR).

| Cluster location | Peak (MNI) | | | Cluster size (voxels) | *t* value |
| --- | --- | --- | --- | --- | --- |
|  | x | y | z |  |  |
| *Seed: left superior temporal gyrus* |  |  |  |  |  |
| Left Cerebellum Crus I | -30 | -72 | -33 | 53 | -4.4019 |
| Left Thalamus | 3 | -18 | 18 | 60 | -4.6192 |
| *Seed: right supplementary motor area* |  |  |  |  |  |
| Right DLPFC | 24 | 57 | 3 | 16 | -4.7556 |
| Right Precuneus | 9 | -63 | 60 | 15 | -4.2985 |

The significant threshold was *P* < 0.05 (Gaussian random field corrected, voxel *P* < 0.001, cluster *P* < 0.05). Age, sex, and the mean FD values were used as covariates to minimize the potential effects of these variables. MNI = Montreal Neurological Institute; DLPFC = dorsolateral prefrontal cortex; OCD = obsessive-compulsive disorder; FD = framewise displacement.

**Table S3.** Brain regions with abnormal dynamic functional connectivity at rest in OCD (window length = 80 TRs, step size = 1 TR).

| Cluster location | Peak (MNI) | | | Cluster size (voxels) | *t* value |
| --- | --- | --- | --- | --- | --- |
|  | x | y | z |  |  |
| *Seed: left superior temporal gyrus* |  |  |  |  |  |
| Left Cerebellum Lobule Ⅵ | -15 | -42 | -39 | 73 | -4.4963 |
| Left Thalamus | -9 | -51 | 15 | 68 | -3.9912 |
| *Seed: right supplementary motor area* |  |  |  |  |  |
| Left Precuneus | 3 | -63 | 33 | 43 | -4.4464 |

The significant threshold was *P* < 0.05 (Gaussian random field corrected, voxel *P* < 0.001, cluster *P* < 0.05). Age, sex, and the mean FD values were used as covariates to minimize the potential effects of these variables. MNI = Montreal Neurological Institute; OCD = obsessive-compulsive disorder; FD = framewise displacement.

**Table S4.** Brain regions with abnormal dynamic functional connectivity at rest in OCD (window length = 50 TRs, step size = 3 TRs).

| Cluster location | Peak (MNI) | | | Cluster size (voxels) | *t* value |
| --- | --- | --- | --- | --- | --- |
|  | x | y | z |  |  |
| *Seed: left superior temporal gyrus* |  |  |  |  |  |
| Left Cerebellum Crus I | -3 | -75 | -33 | 62 | -4.0988 |
| Left Thalamus | 3 | -18 | 18 | 68 | -4.2157 |
| *Seed: right supplementary motor area* |  |  |  |  |  |
| Right DLPFC | 24 | 57 | 3 | 23 | -5.1729 |
| Left Precuneus | 0 | -60 | 36 | 55 | -4.0059 |

The significant threshold was *P* < 0.05 (Gaussian random field corrected, voxel *P* < 0.001, cluster *P* < 0.05). Age, sex, and the mean FD values were used as covariates to minimize the potential effects of these variables. MNI = Montreal Neurological Institute; DLPFC = dorsolateral prefrontal cortex; OCD = obsessive-compulsive disorder; FD = framewise displacement.

**Table S5.** Brain regions with abnormal dynamic functional connectivity at rest in OCD (window length = 50 TRs, step size = 5 TRs).

| Cluster location | Peak (MNI) | | | Cluster size (voxels) | *t* value |
| --- | --- | --- | --- | --- | --- |
|  | x | y | z |  |  |
| *Seed: left superior temporal gyrus* |  |  |  |  |  |
| Left Cerebellum Crus I | -3 | -75 | -33 | 64 | -4.0643 |
| Left Thalamus | 3 | -18 | 18 | 63 | -4.2166 |
| *Seed: right supplementary motor area* |  |  |  |  |  |
| Right DLPFC | 24 | 57 | 3 | 24 | -5.1626 |
| Left Precuneus | 0 | -60 | 36 | 46 | -3.8994 |

The significant threshold was *P* < 0.05 (Gaussian random field corrected, voxel *P* < 0.001, cluster *P* < 0.05). Age, sex, and the mean FD values were used as covariates to minimize the potential effects of these variables. MNI = Montreal Neurological Institute; DLPFC = dorsolateral prefrontal cortex; OCD = obsessive-compulsive disorder; FD = framewise displacement.


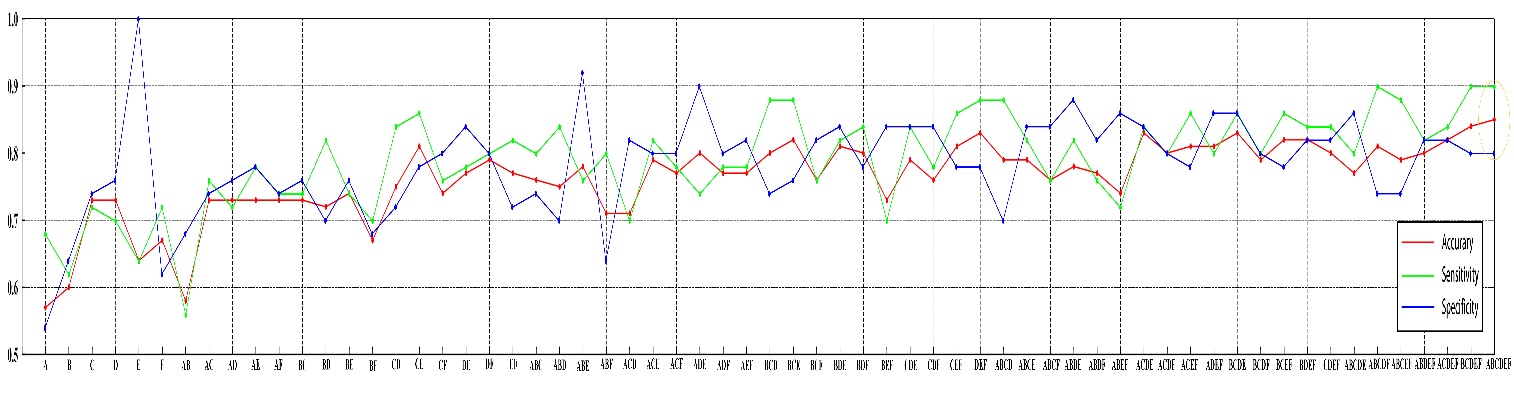


**Fig. S1**. Support vector machine (SVM) results. The combination of brain regions with altered GMV and dFC (features A, B, C, D, E and F) could distinguish OCD from controls accurately. A = GMV of STG; B = GMV of right SMA; C = dFC of left STG-left cerebellum Crus I; D = dFC of left STG-left thalamus; E = dFC of right SMA-right DLPFC; F = dFC of right SMA-left precuneus. STG: superior temporal gyrus; SMA: supplementary motor area; DLPFC: dorsolateral prefrontal cortex; GMV: gray matter volume; dFC; dynamic functional connectivity.


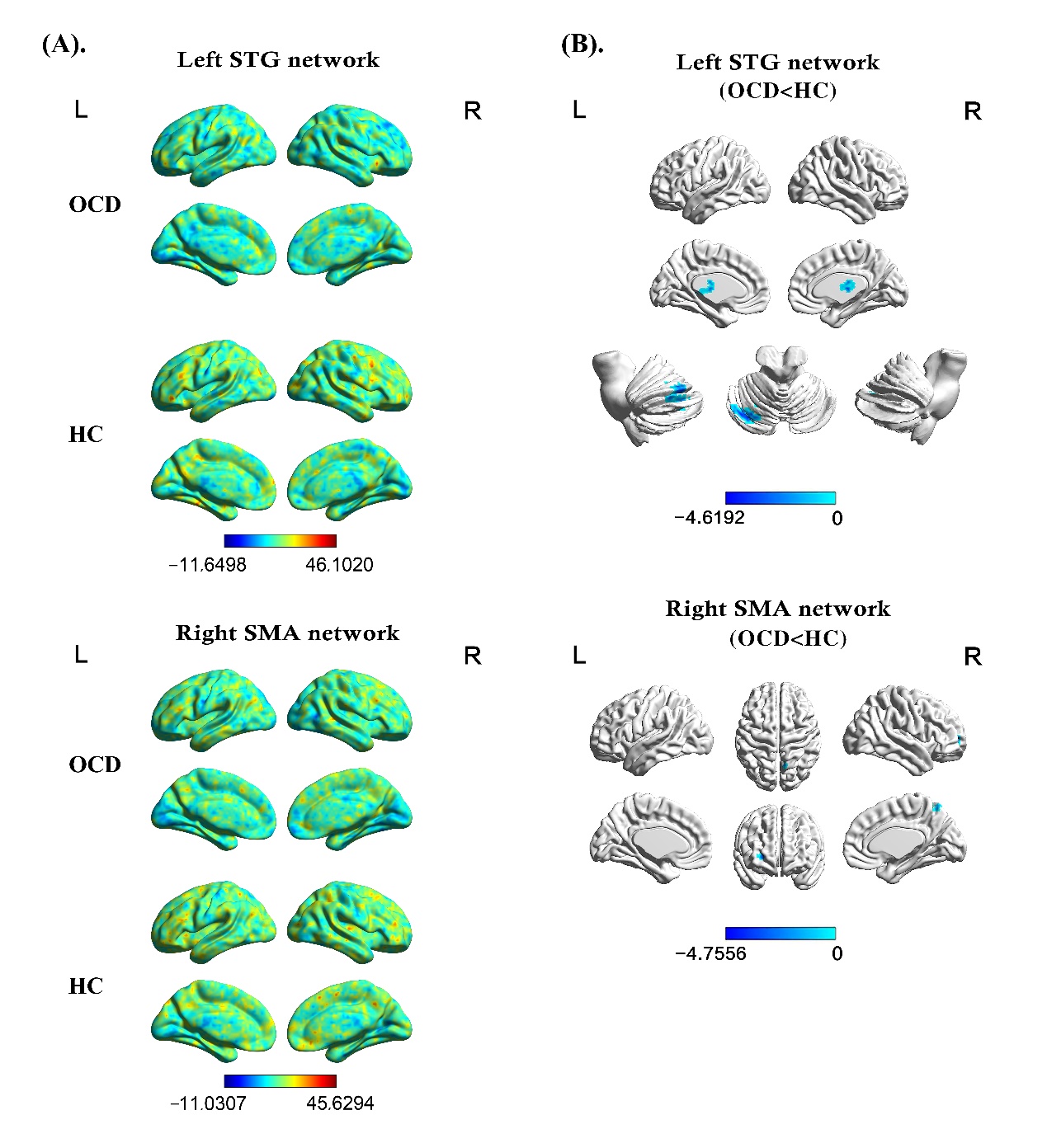


**Fig. S2.** Voxel-wise analysis of dFC patterns in abnormal GMV brain regions (window length = 30 TRs, step size = 1 TR). The color bar indicates the *t* values from one/two-sample *t*-tests. (A) DFC pattern maps of the left STG network and right SMA network in OCD and HC group separately. (B) Brain regions with abnormal dFC in OCD. The blue color denotes decreased dFC values in OCD. STG: superior temporal gyrus; SMA: supplementary motor area; dFC: dynamic functional connectivity; OCD: obsessive-compulsive disorder; HC: healthy control; L: left; R: right.


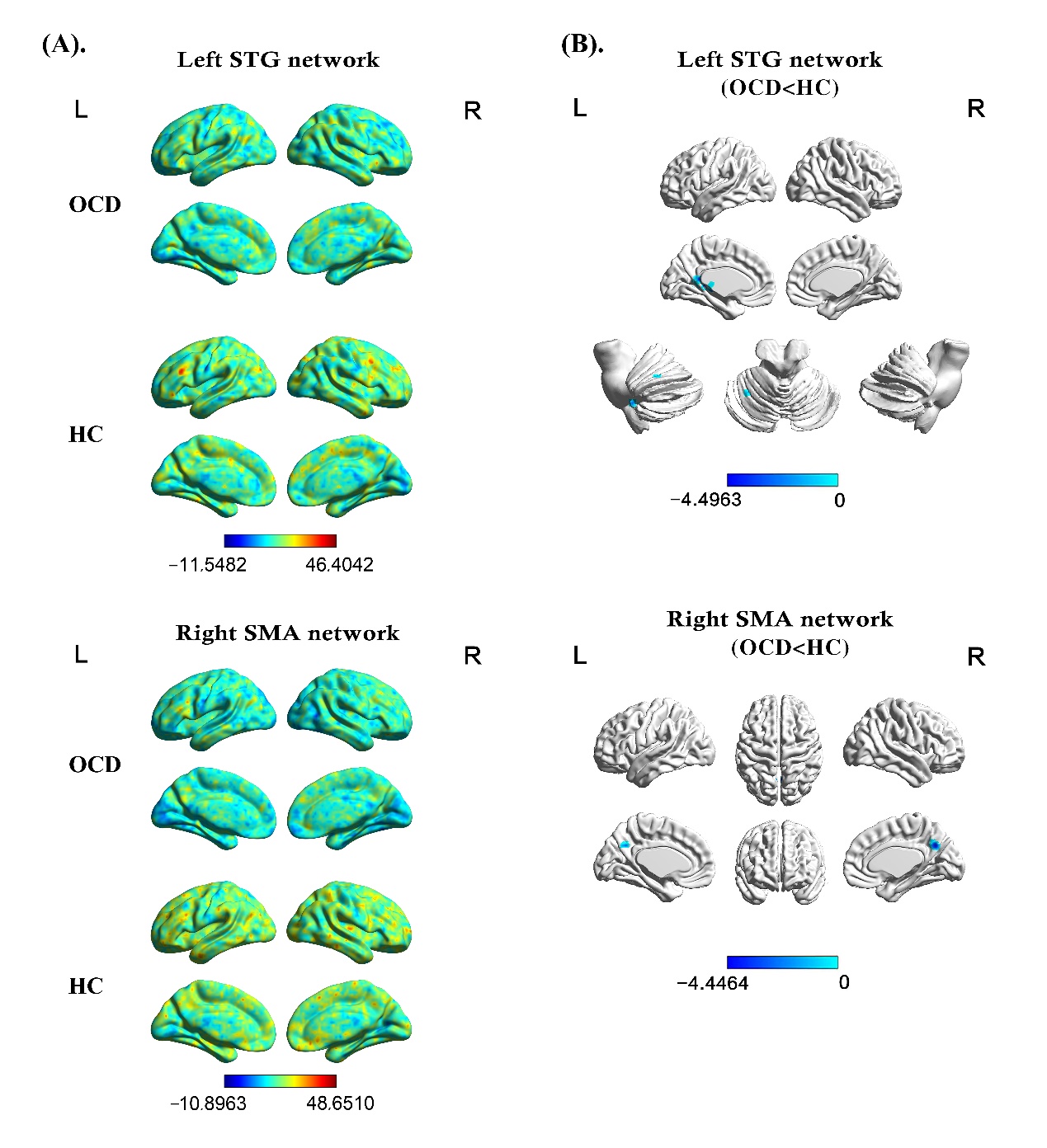


**Fig. S3.** Voxel-wise analysis of dFC patterns in abnormal GMV brain regions (window length = 80 TRs, step size = 1 TR). The color bar indicates the *t* values from one/two-sample *t*-tests. (A) DFC pattern maps of the left STG network and right SMA network in OCD and HC group separately. (B) Brain regions with abnormal dFC in OCD. The blue color denotes decreased dFC values in OCD. STG: superior temporal gyrus; SMA: supplementary motor area; dFC: dynamic functional connectivity; OCD: obsessive-compulsive disorder; HC: healthy control; L: left; R: right.


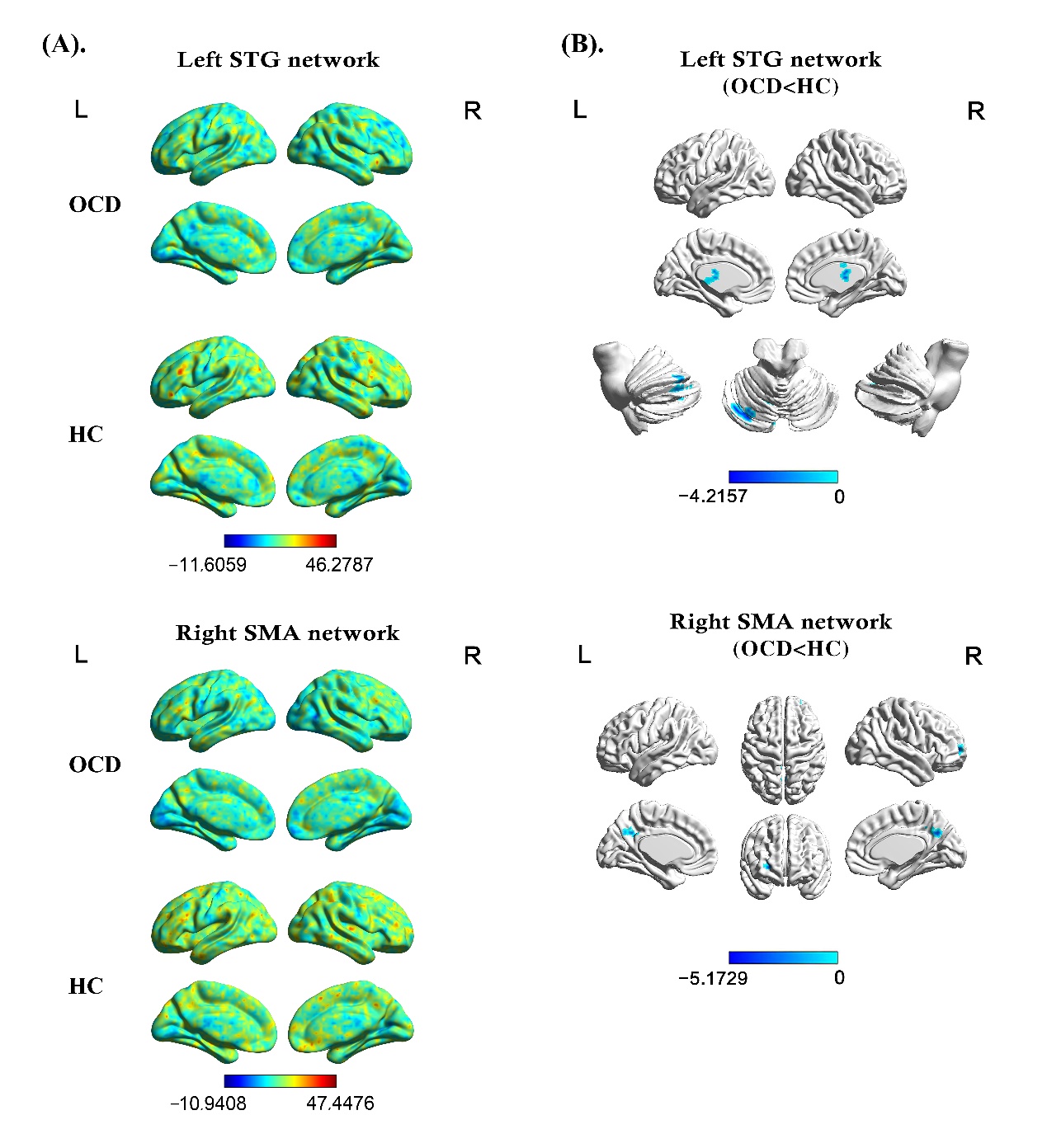


**Fig. S4.** Voxel-wise analysis of dFC patterns in abnormal GMV brain regions (window length = 50 TRs, step size = 3 TRs). The color bar indicates the *t* values from one/two-sample *t*-tests. (A) DFC pattern maps of the left STG network and right SMA network in OCD and HC group separately. (B) Brain regions with abnormal dFC in OCD. The blue color denotes decreased dFC values in OCD. STG: superior temporal gyrus; SMA: supplementary motor area; dFC: dynamic functional connectivity; OCD: obsessive-compulsive disorder; HC: healthy control; L: left; R: right.


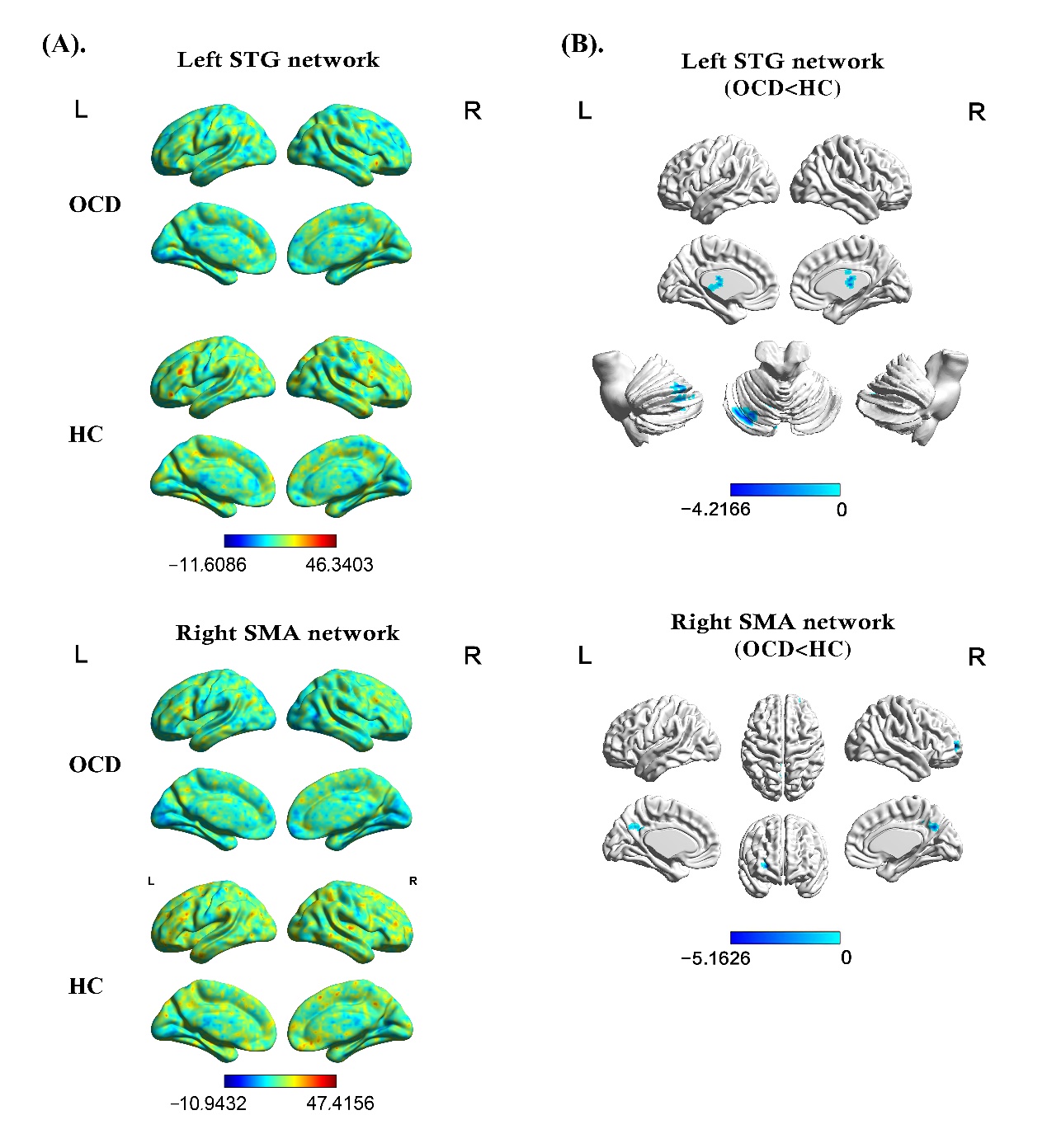


**Fig. S5.** Voxel-wise analysis of dFC patterns in abnormal GMV brain regions (window length = 50 TRs, step size = 5 TRs). The color bar indicates the *t* values from one/two-sample *t*-tests. (A) DFC pattern maps of the left STG network and right SMA network in OCD and HC group separately. (B) Brain regions with abnormal dFC in OCD. The blue color denotes decreased dFC values in OCD. STG: superior temporal gyrus; SMA: supplementary motor area; dFC: dynamic functional connectivity; OCD: obsessive-compulsive disorder; HC: healthy control; L: left; R: right.


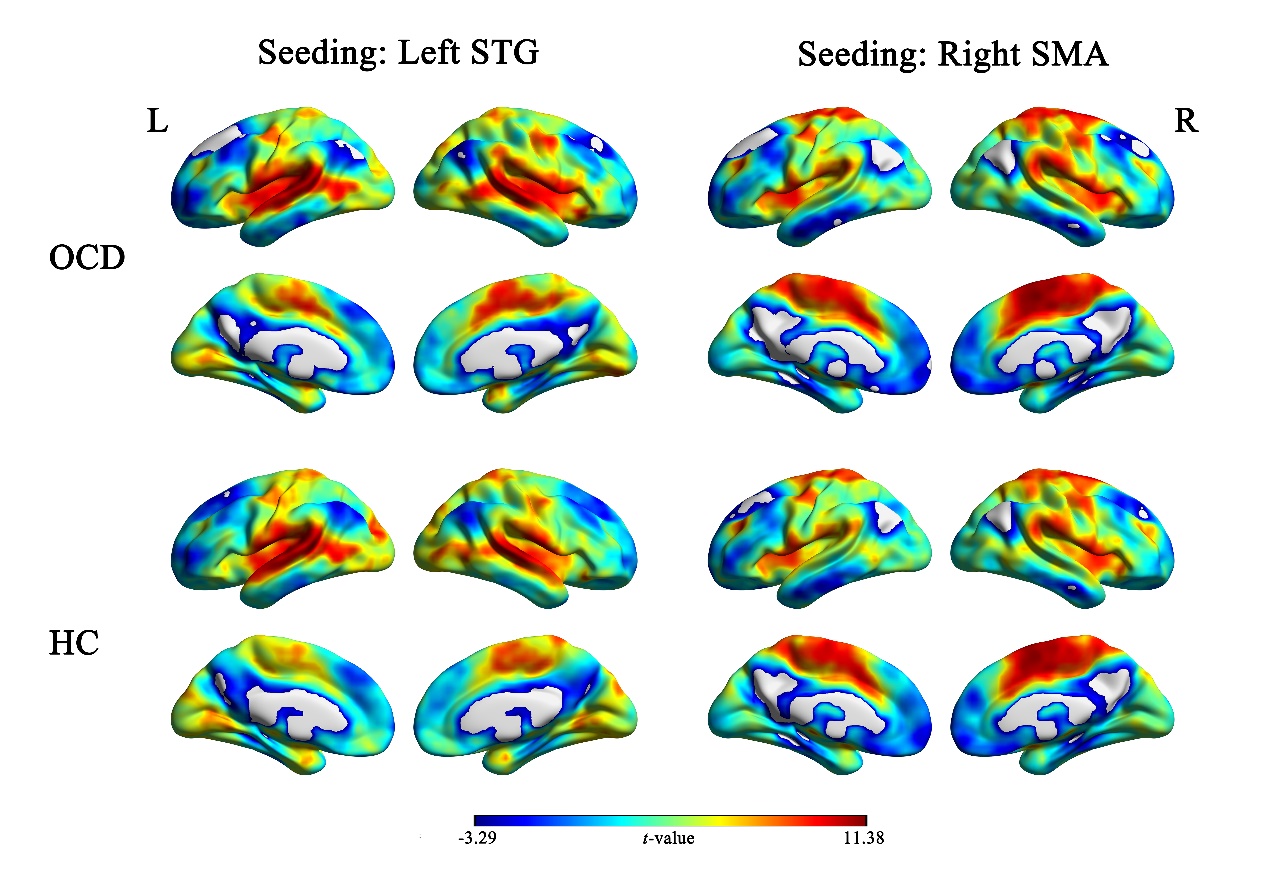


**Fig. S6**. Static functional connectivity at rest in OCD and HC groups. The significance threshold was defined as *P* < 0.05 with Gaussian Random Field (GRF) correction. STG: superior temporal gyrus; SMA: supplementary motor area; OCD: obsessive-compulsive disorder; HC: healthy control; L: left; R: right.
